# Morphological responses of *Arabidopsis thaliana* wild type and photoreceptor genotypes to narrowband UV radiation generated by LEDs

**DOI:** 10.1111/plb.70105

**Published:** 2025-09-17

**Authors:** N. Cunningham, G. Crestani, A. P. Morrison, M. A. K. Jansen

**Affiliations:** ^1^ School of Biological, Earth and Environmental Science & Environmental Research Institute University College Cork Cork Ireland; ^2^ Electrical & Electronic Engineering, School of Engineering & Architecture University College Cork Cork Ireland

**Keywords:** Morphology, narrowband UV, petiole, photoreceptor, UV‐LED, UVR8

## Abstract

- Sensing and responding to light signals are critical factors for plant survival. Plants have photoreceptors which coordinate morphological responses through divergent yet overlapping signalling networks. This study aims to identify how *Arabidopsis thaliana* fine‐tunes its architecture in response to different wavelengths of UV radiation.
- Using narrow wavelength UV‐emitting LEDs, in combination with a set of photoreceptor genotypes, enabled investigation of photoreceptor‐dependent effects on leaf morphology. Eight *Arabidopsis* genotypes, including wildtype Col‐0, *uvr8‐6, cry1, cry2, cry1cry2, phot1, phot2* and *phot1phot*2*,* were exposed to ~45 μW cm^−2^ narrowband UV, with emission peaks at 310, 325, 340 or 365 nm.
- (a) UV‐B wavelengths have a strong inhibitory effect on petiole elongation, with modest effects on leaf blade width and area. (b) Inhibitory effects of narrowband UV‐B on petiole elongation are mediated by UVR8. Phototropins and cryptochromes antagonise this effect, implying that these photoreceptors perceive and mediate responses to UV‐B. (c) Short wavelength UV‐A also induces dwarfing of petioles, but not leaf blades, and this is mediated by UVR8. Cryptochromes impede dwarfing under short wavelength UV‐A. (d) Longer wavelength UV‐A responses are mediated by both phototropins and cryptochromes, with opposing effects on petiole elongation. (e) Although no UVR8‐mediated morphological effect was measured under longer UV‐A wavelengths, UVR8 affects gene expression throughout the UV‐A and UV‐B spectral zones.
- UVR8, cryptochromes and phototropins are all active across all UV‐B and UV‐A wavelengths, controlling multiple interactive morphological and/or gene expression effects.

## Graphical abstract

Arabidopsis photoreceptor mutants exposed to narrowband UV radiation reveal that UVR8, cryptochromes and phototropins are active across both the UV‐B and UV‐A spectrum; regulating leaf morphology and/or gene expression through overlapping yet distinct signalling roles.

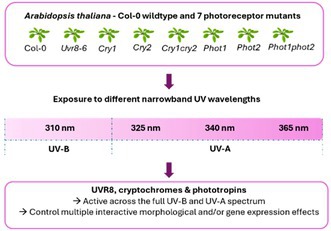

## INTRODUCTION

Phenotypic plasticity is central to ensuring the survival and vigour of plants in response to a range of environmental cues and stressors (Kami *et al*. 2010; Bush 2023). Arguably, light is the most important factor throughout a plant's entire life cycle, as sensing and responding to light signals are critical for survival. Consequently, plants have evolved photoreceptors which coordinate morphological responses through divergent, yet overlapping signalling networks (Parihar *et al*. 2016). At present, five main classes of plant photoreceptors are known, each possessing unique functionalities and absorption spectra (Rai *et al*. 2019). These are phytochromes (red/far‐red), F‐box‐containing Flavin binding proteins such as ZEITLUPE (blue/UV‐A), cryptochromes (blue/UV‐A), phototropins (blue/UV‐A) and UV RESISTANCE LOCUS 8 (UVR8) (UV‐B/shorter wavelength UV‐A) (Rizzini *et al*. 2011; Parihar *et al*. 2016; Paik & Huq 2019; Rai *et al*. 2019).

Photoreceptors are involved in UV‐induced morphological responses, yet their full functionality at specific wavelengths and in various parts of the plant is not yet elucidated. This may, in part, be because in many studies UV‐B is supplied in conjunction with UV‐A, thus creating uncertainty around wavelength‐ and photoreceptor‐specific UV responses. Broadband UV‐B induced phenotypic adjustments have been widely studied, both in the context of UVR8 signalling and general stress responses, referred to as stress‐induced morphogenic responses (SIMR) (Potters *et al*. 2007). In the absence of SIMR, UVR8 mediates photomorphogenic responses to UV‐B radiation, triggering signalling cascades which ultimately culminate in a more compact phenotype with smaller, thicker leaves, reduced root growth, increased axillary branching and shorter petioles (Robson *et al*. 2015; Zhang *et al*. 2019; Crestani *et al*. 2023; Cunningham *et al*. 2024). However, the specific UV‐B wavelengths that drive UVR8‐mediated responses have not been identified. UV‐A‐induced phenotypic effects are under‐researched by comparison. UV‐A‐induced morphological adjustments include changes in leaf area, stem and petiole length and biomass. Adjustments may relate to increases or decreases in expansion growth, thus leading to contradictory reports (Chen *et al*. 2019; Qian *et al*. 2023). The specific UV‐A wavelengths responsible for these phenotypic adjustments have not been conclusively identified. There have been suggestions to subdivide the UV‐A spectrum into two distinct regions: UV‐A1 and UV‐A2. This is based on the perception of UV‐A by photoreceptors and assumes short wavelength UV‐A radiation (315–350 nm, UV‐A2) is absorbed mostly by UVR8, while longer wavelength UV‐A (350–400 nm, UV‐A1) is absorbed by cryptochromes (CRY) and phototropins (PHOT) (Rai *et al*. 2019; Sun *et al*. 2024).

Operating at the centre of photomorphogenic signalling pathways is the COP1 (CONSTITUTIVE PHOTOMORPHOGENIC 1) protein. COP1 forms a complex with SPA proteins to regulate many aspects of plant life (Ponnu & Hoecker 2022). The COP1 complex directly interacts with UVR8 and CRY1/2 in a UV‐B‐, UV‐A‐ and blue light‐dependent manner, destabilising early signalling factors, including phytochrome‐interacting transcription factors (PIFs) PIF4 and PIF5 (Bhatnagar *et al*. 2020). PIFs further relay signals to downstream effectors, such as ELONGATED HYPOCOTYL 5 (HY5), which can bind to the promoters of some 4000 genes, regulating expression of most CRY/UVR8‐associated blue‐ and UV‐responsive genes (Ponnu *et al*. 2019; Bhatnagar *et al*. 2020; Rai *et al*. 2020). In parallel, phototropin signalling cascades effect growth and development in a COP1 independent manner (Thoma *et al*. 2020; Depaepe *et al*. 2023). However, the full mechanism of phototropin signalling pathways remains unresolved (Sullivan *et al*. 2021; Łabuz *et al*. 2022).

This UV‐driven signalling is associated with hormone signalling pathways, resulting in changes in auxins, gibberellins (GA) and brassinosteroids (BR) and, ultimately UV‐B and UV‐A phenotypes (Vanhaelewyn *et al*. 2016). For example, PIF4/5 are involved in both negative and positive feedback loops that enable the equilibration of auxin and BR effects on plant growth and development. PIF4/5 also acts as part of a transcription factor cascade required to promote BR synthesis and hypocotyl elongation in *Arabidopsis*. As part of the cascade, PIF4/5 bind to promoters of key biosynthesis genes, such as DWARF4 (DWF4), to promote their expression and influence growth (Wei *et al*. 2017). Furthermore, it has been shown that both UVR8 and CRYs interact with key BR signalling transcription factors, Bri1‐EMS Suppressor 1 (BES1) and BES1 Interacting MYC‐Like protein (BIM1) (Nolan *et al*. 2017; S. Wang *et al*. 2018a; Liang *et al*. 2019). Upon UV‐B exposure the UVR8‐BES1/UVR8‐BIM1 complex inhibits expression of BR growth‐related genes, impeding hypocotyl elongation (Yin *et al*. 2005; Wang *et al*. 2018a; Liang *et al*. 2019). To add to this complexity, research has shown that plasticity can be highly topical, with different cells of the same organ responding differently to UV radiation (Depaepe *et al*. 2023). Similarly, Cunningham *et al*. (2024) found that broadband UV‐B exerted greater inhibitory effects on petiole tissue than on leaf blades.

The current study aims to identify how *Arabidopsis thaliana* plants fine‐tune their architecture in response to different wavelengths of narrowband UV radiation. Using narrow wavelength UV‐emitting LEDs, in combination with a set of well characterised photoreceptor genotypes, enabled investigation of photoreceptor‐dependent effects on leaf morphology. UV responses of specific parts of the leaf were characterised. Utilising petioles of Col‐0 and *uvr8‐6*, the potential role of UVR8, and key transcription factors and genes in UV phototropic responses were assessed.

## MATERIAL AND METHODS

### Plant material

Eight *A. thaliana* genotypes, including wild type Columbia‐0 (Col‐0), *uvr8‐6, cry1, cry2, cry1cry2, phot1, phot2* and *phot1phot2* were utilised in this study (Table S1). All plants used were in the wild type Col‐0 background.

### Growth conditions

Seeds were placed on damp filter paper in Petri dishes and refrigerated at 4°C for 72 h. Following stratification, seeds were transferred to moist jiffy plugs, placed in plastic trays, covered with cling film, and moved to a controlled growth room (Deker Horticulture, Co. Meath, Ireland). Plants were subject to a 14‐h light/10‐h dark photoperiod, provided by cool white photosynthetically active radiation (PAR) tubes (Philips, master TL5 HE 28 W/840 SLV/40. Eindhoven, The Netherlands). PAR intensity was 55 μmol m^−2^ s^−1^. Temperature and relative humidity were set to 22°C and 60%, respectively.

Once seeds had germinated, the cling film was removed, and plants were thinned, leaving one seedling per plug. Plants were grown for ca. 16 days until they developed four rosette leaves >1 mm long, corresponding to growth stage 1.04, as defined by Boyes *et al*. (2001). Plants were watered with distilled water two to three times per week.

### UV exposure conditions

Experiments were conducted using five custom‐built LED lamps (University College Cork, Ireland). Each lamp had both red and blue LEDs (Roithner Electronics, Austria) delivering PAR at an intensity of ~33 ± 2 μmol m^−2^ s^−1^. Four of the lamps contained single wavelength UV LEDs with emission peaks at 310 nm, 325 nm, 340 nm, or 365 nm (DUVXXX series, 120° viewing angle; Roithner Electronics). These wavelengths were chosen because of the match with the absorption maxima of effected photoreceptors: UVR8 ~ 280 nm, CRY1 and CRY2 absorption peaks ~370 nm and 450–500 nm for UV‐A and blue regions, respectively, with CRY1 absorbing also in the UV‐B range; PHOT1 and PHOT2 absorption peaks ~370–380 nm and ~450 nm, respectively, for UV‐A and blue region, with PHOT1 additionally showing UV‐B absorption peaks at 295 and 315 nm (Kasahara *et al*. 2002; Brown *et al*. 2009; Liu *et al*. 2011; Díaz‐Ramos *et al*. 2018; Vanhaelewyn *et al*. 2020). *In planta* absorption differs due to optical properties of the leaves; for instance, UVR8 has an absorption peak at 280 nm *in‐vitro*, yet maximum UV‐B photon effectiveness *in planta* occurs between 290 and 300 nm (Brown *et al*. 2009; Díaz‐Ramos *et al*. 2018). Therefore, wavelengths utilised in this study are conducive to exploring functional response of photoreceptors *in‐vivo*.

Each lamp was set to an intensity of ~46 ± 2 μW cm^−2^ to determine plant responses across different UV wavelengths. UV was measured using a spectroradiometer (Flame‐S, Ocean Optics) and associated software (Oceanview v. 1.6.7). Using the measured spectral irradiance in conjunction with biological spectral weighting functions as described by Flint & Caldwell (2003), the daily biologically effective doses were calculated (310 nm: 1.641 kJ m^−2^; 325 nm: 0.242 kJ m^−2^; 340 nm: 0.238 kJ m^−2^; 365 nm: 0.171 kJ m^−2^). On day 17 of growth, plants were moved under lamps where they received 14 h PAR form 07:00–21:00 h. Additionally, plants under UV lamps were primed with a specific UV‐B/UV‐A wavelength from 10:00–11:30 h (1.5 h). Thereafter, they received 6 h UV (10:00–16:00 h) for the duration of the experiment, which was 8 days (inclusive of the priming day).

### Morphological analyses

On the final day of UV exposure, rosettes were dissected by separating each leaf individually according to developmental order (Nguyen & McCurdy 2015). Leaves were photographed, and images analysed using ImageJ v. 1.53c software (Wayne Rasband, National Institutes of Health, USA). Cotyledons were not included, nor were leaves with a petiole length <2 mm. Accordingly, the six oldest measurable leaves per plant were included in the analysis, and the following parameters were assessed: leaf blade length, width, area and petiole length. In cases where younger, measurable leaves were present, they were excluded so as to standardise the procedure. The experiment was independently replicated three times, with each replicate comprising three plants per treatment.

### Gene expression analysis in petioles

#### Total RNA extraction and cDNA synthesis

Immediately after cessation of UV treatments, petioles from a minimum of 15 plants were pooled, ground to a fine powder in liquid nitrogen and placed in a −80°C freezer for gene expression analyses. RNA was then isolated using the RNeasy plant mini kit (Qiagen, Manchester, UK) including an on‐column DNA digestion step (RNase Free DNase Set; Qiagen) following the manufacturer's specifications. RNA concentration and purity were assessed using a NanoDrop 1000 Spectrophotometer (Thermo Fisher Scientific, Waltham, MA, USA). Integrity of RNA was determined by running an aliquot of each sample on a 1% denaturing agarose gel, stained with ethidium bromide (EtBr). In detail, 2 μL RNA, 10 μL formamide, 3 μL formaldehyde, 2.5 μL MEN buffer and a drop of EtBr were heated at 65°C for 10 min. Samples were loaded on an agarose gel and products visualised with a transilluminator. cDNA was synthesised following reverse transcription with the NZY First Strand cDNA Synthesis Kit (MB12501; NZYTech, Lisbon, Portugal) according to the manufacturer's protocol.

#### Testing primer efficiency of genes of interest

Primer sequences of a reference gene and eight genes of interest were obtained from publications or designed using Primer3 and purchased from IDT (Integrated DNA Technologies, Leuven, Belgium). Primers were tested by amplifying cDNA and running products on a 1% gel, stained with SYBR safe (1 μL per 10 μL agarose gel). Only primers with an efficiency >80% were used for quantitative real‐time PCR (Table S2).

#### Gene expression analysis

Gene expression analysis was carried out using a Real‐Time PCR System (7500; Applied Biosystems). A volume of 1 μL diluted cDNA (1:4) was mixed with forward and reverse primer (0.4 μL each), 3.2 μL water and 5 μL Applied Biosystems SYBR Select Master Mix (Applied Biosystems). Quantitative Real‐Time PCR conditions consisted of an initial holding stage of 2 min at 50°C, and a 10 min ramp to 95°C. This was followed by 40 cycles of 15 s at 95°C and 1 min at 60°C. The melt curve stage consisted of an initial phase of 15 s at 95°C, 1 min at 60°C, a 30 s ramp to 95°C and a 15 s decrease to 60°C. Each sample was analysed in triplicate and results were obtained from two biological replicates. Relative gene expression levels of target genes were calculated using the Pfaffl equation and fold change was also calculated (Pfaffl 2001).

### Statistical analysis

**Fig. 1:**
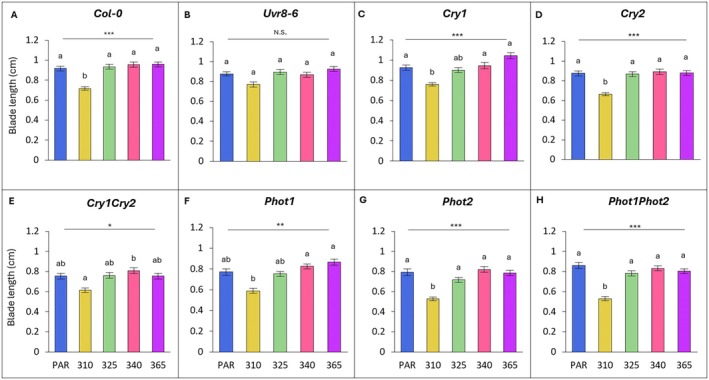
Blade length across wavelength, grouped by genotype (A–H). Bars (± SE) represent mean blade length of three independent replicates. Bars represent different wavelengths: blue bar = PAR control, yellow = 310 nm, green = 325 nm, pink = 340 nm, purple = 365 nm. For individual genotypes, blade length was compared across wavelengths (one‐way ANOVA or Kruskal‐Wallis); asterisks above the line indicate level of significance of the overall model, with *P* < 0.05 (*), *P* < 0.01 (**) or *P* < 0.001 (***); N.S. denotes not significant. For each genotype, light treatments with a different letter are statistically different from each other (post‐hoc analysis).

**Fig. 2:**
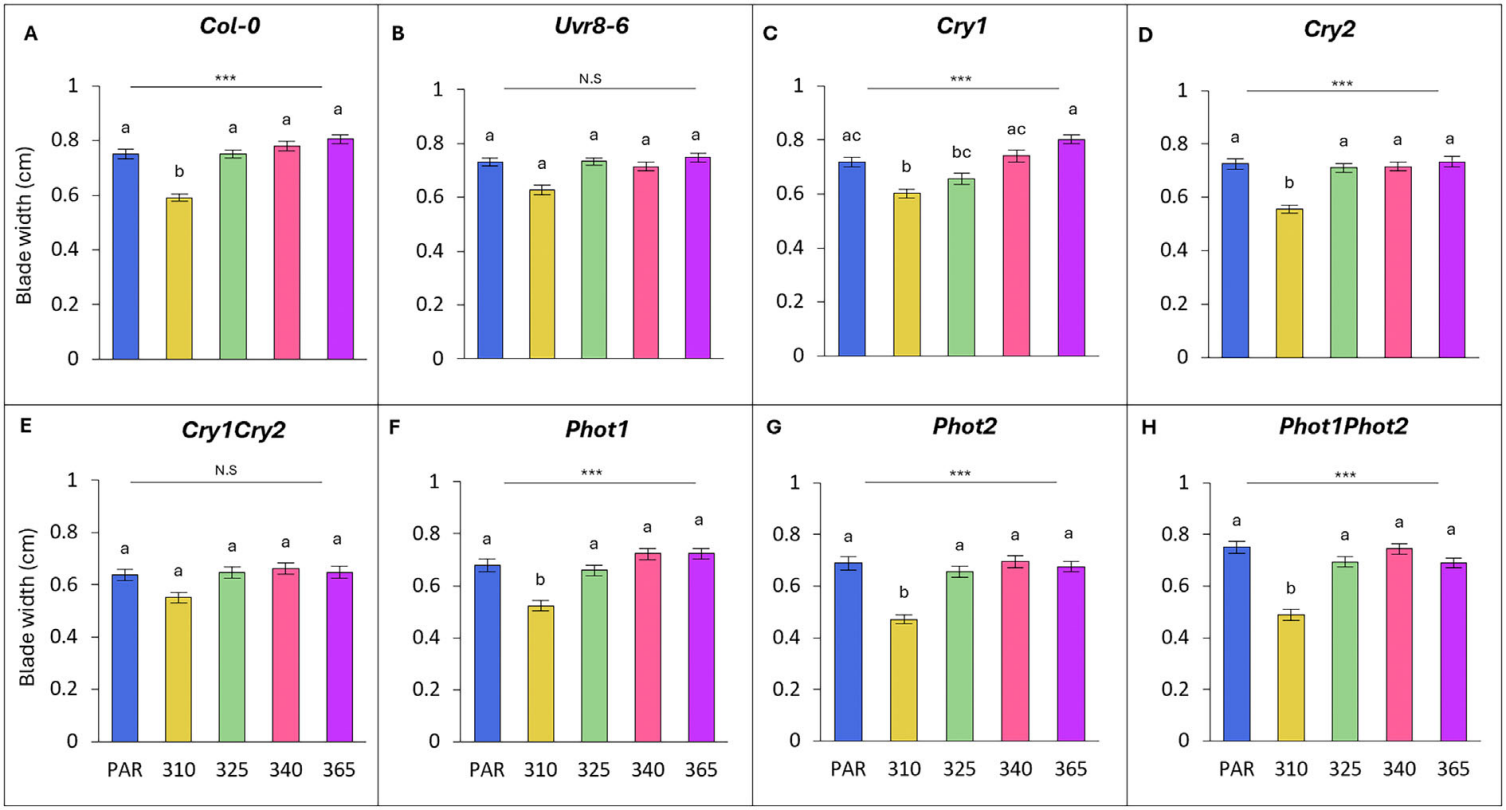
Blade width across wavelength, grouped by genotype (A–H). Bars (± SE) represent mean blade length of three independent replicates. Bars represent different wavelengths: blue = PAR control, yellow = 310 nm, green = 325 nm, pink = 340 nm, purple = 365 nm. For individual genotypes, blade length was compared across wavelengths (one‐way ANOVA or Kruskal‐Wallis); asterisks above the line indicate significance of the overall model, with *P* < 0.05 (*), *P* < 0.01 (**) or *P* < 0.001 (***); N.S. not significant. For each genotype, light treatments with a different letter are statistically different from each other (post‐hoc analysis).

**Fig. 3:**
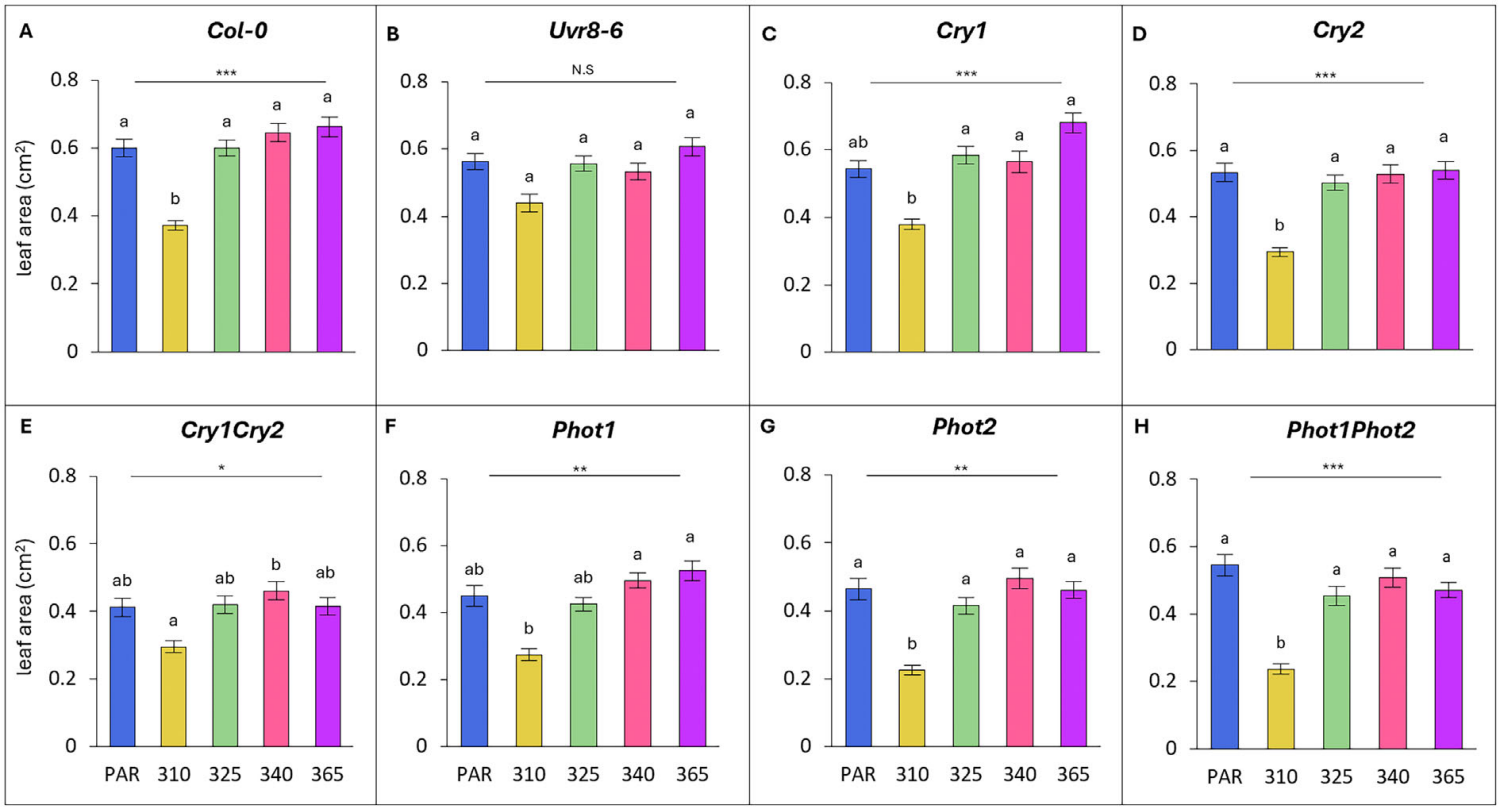
Blade area across wavelength, grouped by genotype (A–H). Bars (± SE) represent mean blade length of three independent replicates. Bars represent different wavelengths: blue bar = PAR control, yellow = 310 nm, green = 325 nm, pink = 340 nm, purple = 365 nm. For individual genotypes, blade length was compared across wavelengths (one‐way ANOVA or Kruskal‐Wallis); asterisks above the line indicate level of significance of the overall model, with *P* < 0.05 (*), *P* < 0.01 (**) or *P* < 0.001 (***); N.S. not significant. For each genotype, light treatments with a different letter are statistically different from each other (post‐hoc analysis).

**Fig. 4:**
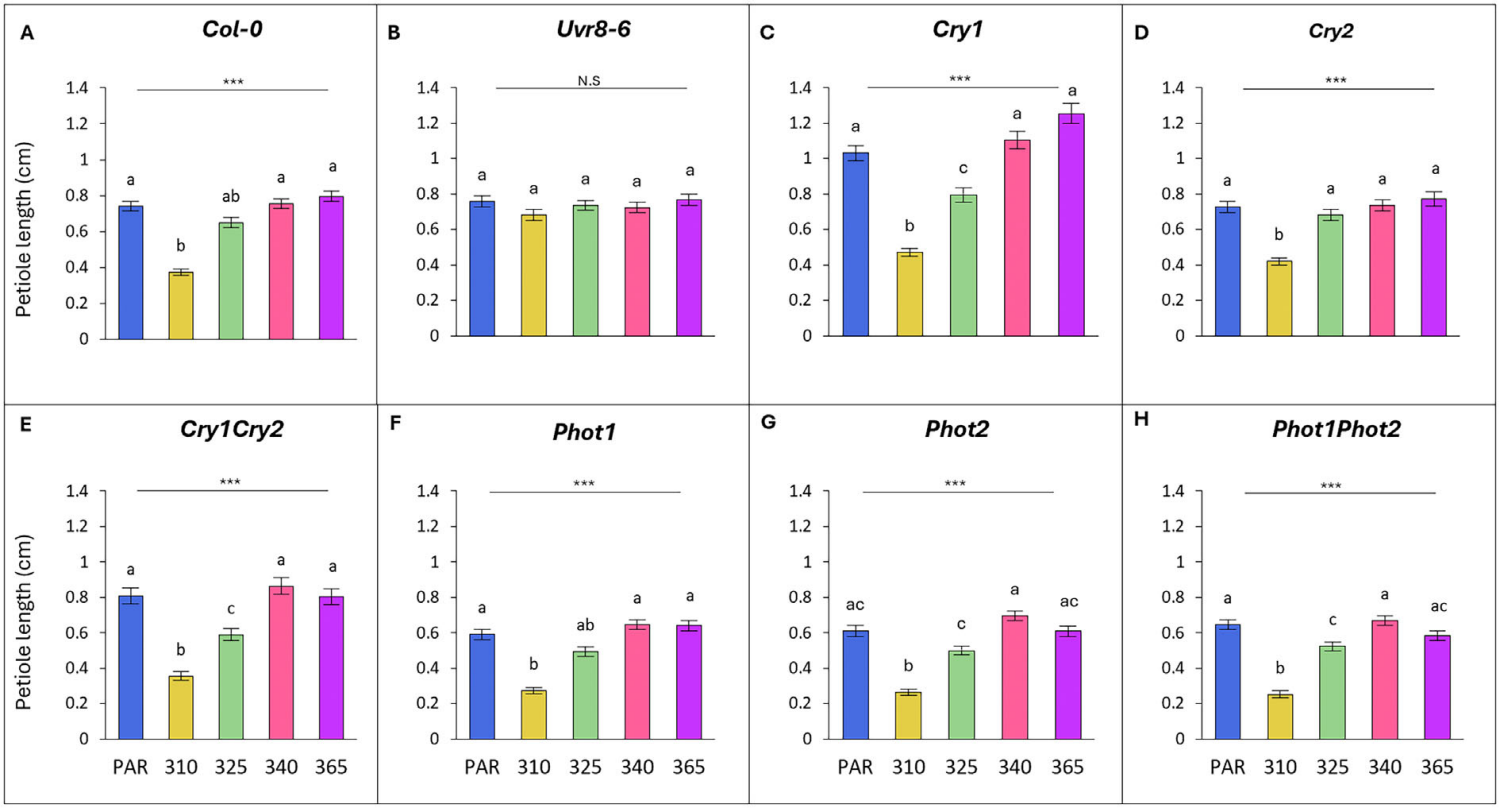
Petiole length across wavelength, grouped by genotype (A–H). Bars (±SE) represent mean blade length of three independent replicates. Bars represent different wavelengths: blue = PAR control, yellow = 310 nm, green = 325 nm, pink = 340 nm, purple = 365 nm. For individual genotypes, blade length was compared across wavelengths (one‐way ANOVA or Kruskal‐Wallis); asterisks above the line indicate level of significance of the overall model, with *P* < 0.001 (***) or N.S. not significant. For each genotype, light treatments with a different letter are statistically different from each other (post‐hoc analysis).

Statistical analyses, including test assumptions, were performed using IBM^®^ SPSS^®^ Statistics v. 28.0 software (Armonk, New York, USA). To analyse the role of the genotype in controlling plant morphology, morphological UV responses of each genotype were assessed independently across all UV wavelengths and compared to respective PAR controls using either one‐way ANOVA or Kruskal‐Wallis tests. The overall statistical significance of the model is reported, together with post‐hoc tests and/or pairwise comparisons using the Bonferroni correction, which shows differences between light treatments (Figs. 1, 2, 3, 4). Additionally, quantitative morphological responses of different genotypes to the same UV wavelength were compared. To do so, morphological data for each UV‐exposed genotype were normalised against growth of the respective PAR control. Normalised growth responses of each genotype were then compared to those of the Col‐0 wild type using either Student's *t*‐tests or Mann‐Whittney *U* tests (Tables 1 and 2; Tables S3 and S4). For relative gene expression data, Col‐0 and *uvr8‐6* were independently assessed across all UV treatments compared to respective PAR controls using either one‐way ANOVA or Kruskal‐Wallis tests, with post‐hoc tests, determining differences between treatments. Furthermore, two‐tailed *t*‐tests were used to identify significant differences between Col‐0 and *uvr8‐6* for each UV treatment. Data are reported as mean ± SE.

## RESULTS

### Leaf blade length

Across all wavelengths, leaf blade lengths of all genotypes, except *uvr8‐6*, were significantly affected by UV treatment relative to their PAR controls (Fig. 1A–H).

In UV‐B (310 nm) exposed Col‐0, blades were 22% shorter compared to the PAR control (*P* < 0.001; Fig. 1A). The *phot1phot2* mutants were most impacted by UV‐B exposure and had blades that were shortened by 38% compared to the PAR control (*P* < 0.001; Fig. 1H). A comparison of relative UV‐B responses showed that leaf blades of *phot1phot2* shortened significantly more than those of Col‐0 (*P* = 0.003; Table S3).

Short wavelength UV‐A, with a peak at 325 nm, did not significantly affect the leaf blade length of any genotypes relative to the PAR control (Fig. 1). However, UV‐A (325 nm) induced a significant decrease in blade length in the *phot1phot2* genotype compared to Col‐0 (*P* = 0.007; Table S3). At longer wavelength UV‐A (340 nm), the absence of phototropins had a negative effect on blade length, as evidenced by significant differences between blade lengths of *phot1phot2* (slightly decreased length) and Col‐0 (slightly increased length) (*P* = 0.034; Table S3). Longer wavelength UV‐A (365 nm) induced slight elongation of Col‐0 leaf blades, yet lengthening effects were most pronounced and significantly larger in *cry1* plants compared to Col‐0 (*P* = 0.018; Table S3).

### Leaf blade width

Across all wavelengths, the leaf blade width of Col‐0, *cry1, cry2, phot1, phot2* and *phot1phot2* were all significantly affected by UV, but this was not the case for *uvr8‐6* and *cry1cry2* (Fig. 2A–H).

Under UV‐B at 310 nm, leaves of Col‐0 plants were 21% narrower compared to PAR controls (*P* < 0.001; Fig. 2A). However, the most impacted were the phototropin‐impaired genotypes *phot1, phot2* and *phot1phot2*, in which blades were 23%, 32% and 35% narrower than the PAR control, respectively (*P* = 0.01; *P* = 0.003; *P* < 0.001; Fig. 2F–H). When compared with Col‐0, this decrease in width was significantly larger in *phot2* and *phot1phot2* genotypes (*P* = 0.029; *P* = 0.01; Table S4) respectively. Furthermore, *cry1* and *cry1cry2* were significantly different to Col‐0 (*P* = 0.041; *P* = 0.013; Table S4).

Under the shorter UV‐A wavelength of 325 nm, leaves were significantly wider than those at 310 nm for all genotypes except for *cry1* and *cry1cry2* (Fig. 2C–E). Compared to Col‐0, a significant relative reduction in width was observed in *cry1* and *phot1phot2* plants (*P* = 0.015 and *P* = 0.026, respectively; Table S4). At UV‐A wavelengths of 340 nm and 365 nm, Col‐0 showed slight leaf broadening (Fig. 2A). However, *cry1* plants had the widest leaves under 365 nm (12%), while *phot1phot2* plants had narrowed leaves under 365 nm (−8%; Table S4).

### Leaf blade area

Across all UV wavelengths changes in leaf blade area for all genotypes, except *uvr8‐6*, were statistically significant (Fig. 3A–H). Findings relate to the cumulative effect of changes in leaf blade length and width and were dependent on wavelength applied.

For Col‐0, leaves exposed to 310 nm displayed a 38% reduction in leaf area (*P* = 0.017; Fig. 3A). Leaf area of *phot1phot2* was most negatively affected by UV‐B at 310 nm, both relative to the PAR control (−57%; Fig. 3H) and compared to Col‐0 (*P* = 0.004; Table 1). *Cry1* was also significantly different to Col‐0 (*P* = 0.045; Table 1).

**Table 1:** Relative change in leaf blade area of each genotype compared to relative change in blade area of Col‐0 (%).

relative leaf area (%)								
	Col‐0	*Uvr8‐6*	*Cry1*	*Cry2*	*Cry1Cry2*	*Phot1*	*Phot2*	*PhotPhot2*
310 nm	−38%	−22%	−30%*	−45%	−28%	−39%	−51%	−57%**
325 nm	0%	−1%	8%	−6%	2%	−5%	−11%	−17%*
340 nm	8%	−5%	4%	−1%	12%	10%	7%	−7%*
365 nm	10%	8%	25%*	1%	1%	17%	−1%	−14%

Values in bold indicate significant differences in responses of genotype to those of Col‐0 wild type. Level of significance denoted with asterisks: *P* < 0.05 (*) or *P* < 0.01 (**). The heatmap shows relative changes in relative leaf area across genotypes, with darker green indicating strongest positive responses, lighter shades of green indicating moderate to minor positive resposes, yellow to darker yellow showing minor to moderate negative responses, orange showing stronger negative responses and darker red indicating the strongest negative responses.

The Col‐0 leaf area was unaffected by UV‐A at 325 nm, but increased by 8% and 10% under 340 nm and 365 nm, respectively, compared to PAR (Fig. 3A). In contrast, leaf area of *phot1phot2* was negatively affected by all UV‐A wavelengths compared to the PAR control, and resulted in leaves that were significantly smaller compared to Col‐0 for both 340 and 365 nm (*P* = 0.016; *P* = 0.012 respectively; Table 1). Overall, relative to PAR controls, *cry1cry2* had the largest increase in leaf area under 340 nm (12%) (Fig. 3E), while *cry1* plants displayed the largest increase under 365 nm (25%) (Fig. 3C) which was significantly larger than the increases in area of Col‐0 leaves under 365 nm (*P* = 0.023; Table 1).

### Petiole length

Across all wavelengths, petioles of all genotypes, apart from *uvr8‐6*, were significantly impacted by UV treatments compared to PAR controls (Fig. 4A–H).

Across all genotypes, apart from *uvr8‐6*, UV‐B at 310 nm had a significant inhibitory effect on petioles (Fig. 4). For Col‐0, this led to a 50% decrease in length under UV‐B compared to PAR (*P* = 0.002; Fig. 4A). *Uvr8‐6* petioles were the least affected of all genotypes relative to PAR (−10%; *P* = 0.57; Fig. 4B), thus had significantly longer petioles compared to Col‐0 (*P* < 0.001; Table 2). *Phot2* and *phot1phot2* plants displayed the most inhibited petioles of all genotypes compared to PAR (−57%, −61%, respectively; both *P* < 0.001), which resulted in greater inhibition compared to Col‐0 (*P* = 0.036; *P* = 0.001, respectively; Table 2). Moreover, petioles of *cry2*, *cry1cry2* and *phot2* were significantly different to those of Col‐0 (*P* = 0.012; *P* = 0.015; *P* = 0.036, respectively; Table 2).

**Table 2:** Relative change in petiole length of each genotype compared to relative change in Col‐0.

relative petiole length (%)								
	Col‐0	*Uvr8‐6*	*Cry1*	*Cry2*	*Cry1Cry2*	*Phot1*	*Phot2*	*PhotPhot2*
310 nm	−50%	−10%***	−54%	−42%*	−56%*	−54%	−57%*	−61%**
325 nm	−12%	−3%*	−23%*	−6%	−27%**	−17%	−18%	−19%
340 nm	2%	−5%	7%	1%	7%	10%*	14%*	3%
365 nm	8%	1%	22%*	6%	−1%*	9%	0%	−10%*

Values in bold indicate responses of genotype are significantly different to those of Col‐0 wild type. Level of significance denoted with asterisks where *P* < 0.05 (*), *P* < 0.01 (**) or *P* < 0.001 (***). The heatmap shows relative changes in relative petiole length across genotypes, with darker green indicating strongest positive response, lighter shades of green indicating moderate to minor positive responses, yellow to darker yellow showing minor to moderate negative responses, orange showing stronger negative responses and darker red indicating the strongest negative responses.

Inhibition of petiole growth persisted under shorter wavelength UV‐A at 325 nm for all genotypes relative to the PAR control, with *cry1, cry1cry2* and *phot1phot2* petioles being significantly reduced (*P* = 0.029; *P* = 0.039; *P* = 0.042 respectively; Fig. 4C, E, H). In addition, the inhibition of *cry1* and *cry1cry2* petioles was significantly larger than that of Col‐0 (*P* = 0.045; *P* = 0.002; Table 2).

UV‐A at 340 nm did not significantly affect petiole length of any genotype relative to the PAR control (Fig. 4). However, petioles of *phot1* and *phot2* mutants had the greatest relative increase overall, which was in fact larger than Col‐0 (*P* = 0.048 and *P* = 0.015 respectively; Table 2). UV‐A, with a peak at 365 nm, induced lengthening in some genotypes, such as *cry1* where petioles were 22% longer, while in *phot1phot2* it had the opposite effect and petioles decreased in length by 10% (Fig. 4C, H). When compared with Col‐0, these effects were statistically significant, and *cry1* petioles were significantly longer than those of Col‐0, while *phot1phot2* petioles were significantly shorter (*P* = 0.017 and *P* = 0.013 respectively; Table 2).

Overall, the petiole UV response differed from the leaf blade response, and this was influenced by both wavelength and genotype.

### Gene expression analysis of key genes in petioles

**Fig. 5:**
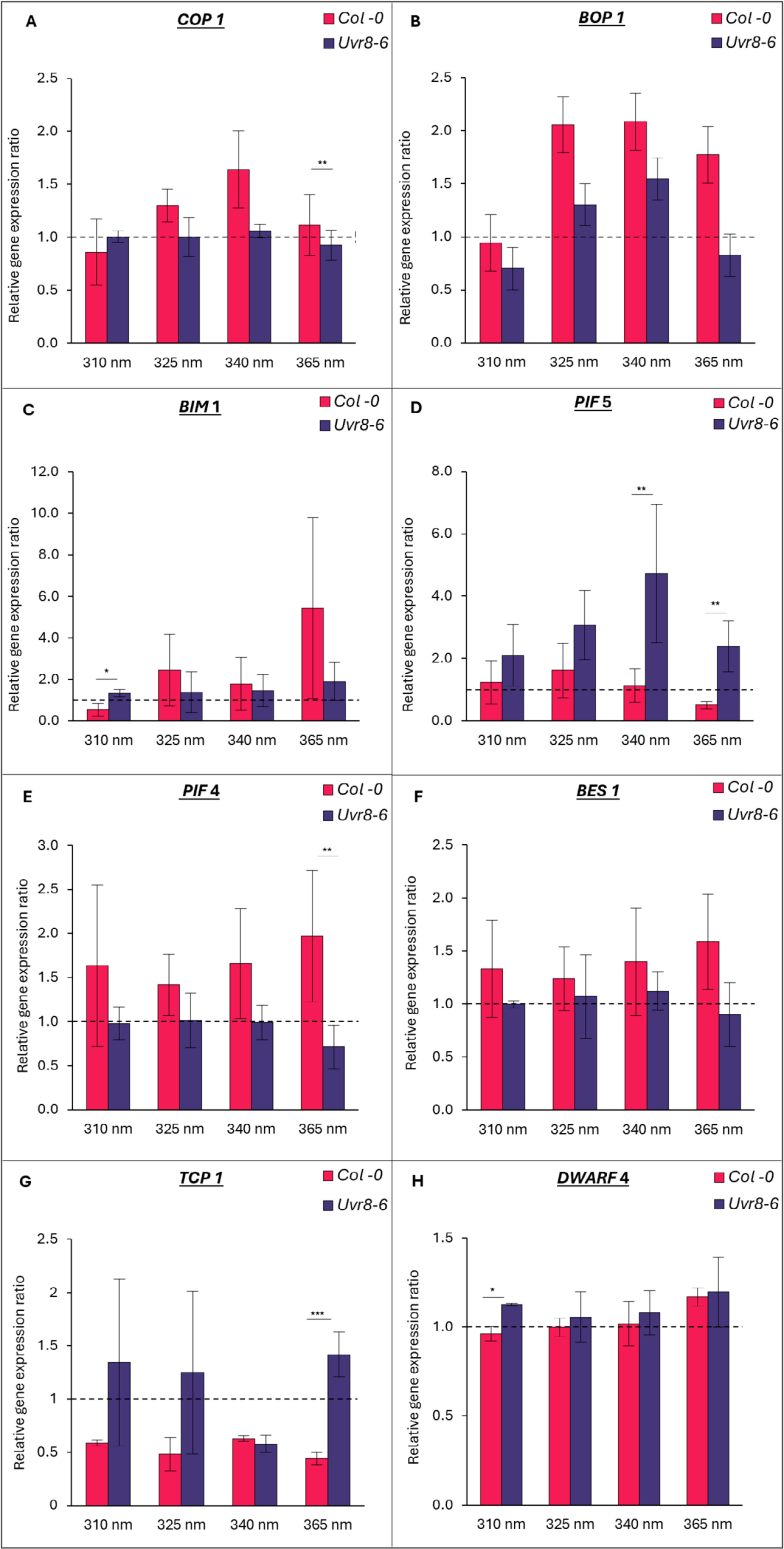
Relative expression of genes hypothesised to be involved petiole length of UV irradiated plants; A) *COP1*, B) *BOP1*, C) *BIM1*, D) *PIF5*, E) *PIF4*, F) *BES1*, G) *TCP1*, H) *DWARF4*. Pink bars represent Col‐0 and purple bars represent uvr8‐6. Genotypes were compared for each treatment separately at 310, 325, 340 and 365 nm. Dashed line represents normalised values of control plants exposed to PAR for each genotype. For each wavelength, asterisks indicate a significant difference between genotypes. *P* < 0.05 (*), *P* < 0.01 (**) or *P* < 0.001 (***). Error bars represent ± SE based on means of three independent replicates.

Relative gene expression analysis was carried out on genes hypothesised to be associated with controlling length of UV‐irradiated petioles. Relative gene expression across UV treatments compared with the PAR control was separately assessed for both Col‐0 and *uvr8‐6* (Table S5; statistically significant data shown). Additionally, the effect of UVR8‐mediated signalling on expression of genes of interest was determined by comparing Col‐0 with *uvr8‐6* at each wavelength (Fig. 5A–H). Across all wavelengths, expression of *COP1* was not significantly affected by UV treatment compared to PAR for either Col‐0 or *uvr8*‐6. Overall, relative expression levels of *COP1* were higher in Col‐0 compared to *uvr8‐6* and this difference was significant at 365 nm (*P* = 0.005, Fig. 5A).

Expression of the key transcription factors *PIF4* and *PIF5* was differently affected by UV treatments for both genotypes (Fig. 5D, E). In Col‐0, *PIF4* expression was significantly upregulated by UV (*P* = 0.045; Table S5), and post‐hoc analysis revealed that expression levels were highest at the longest wavelength of UV‐A (365 nm; two‐fold increase; *P* = 0.02, Table S5). Conversely, relative expression of *PIF4* in *uvr8‐6* petioles was unaffected compared to control conditions. The *t*‐tests comparing genotypes revealed petioles of Col‐0 had higher expression of *PIF4* than *uvr8‐6* and this was significant at 365 nm (*P* = 0.002, Fig. 5C).

The opposite trend was observed for *PIF5*, whereby relative expression was higher in *uvr8‐6* petioles compared to Col‐0, and this difference was significant at 365 nm (*P* = 0.004; Fig. 5D). Indeed, compared to the PAR control, UV treatments enhanced relative expression of *PIF5* for *uvr8‐6* by 2.10‐, 3.07‐, 4.73‐, 2.38‐fold at 310, 325, 340 and 365 nm, respectively (*P* < 0.001; Table S5). In contrast, relative *PIF5* expression in Col‐0 remained unaffected (Table S5).

The expression of *TCP1* in petioles was significantly affected by UV treatments for both Col‐0 and *uvr8‐6* compared to the respective PAR treatments (*P* < 0.001; *P* = 0.002, respectively; Table S5). *TCP1* expression was downregulated in Col‐0 petioles at all wavelengths, and was lowest under 365 nm UV‐A (0.44‐fold decrease; *P* = 0.007; Table S5). Conversely, for *uvr8‐6*, *TCP1* expression was highest under 365 nm, with a 1.4‐fold increase in expression compared to PAR (*P = 0.002*; Table S5). Overall, relative expression of *TCP1* was higher in *uvr8‐6* and was significantly higher compared to Col‐0 at 365 nm (*P* < 0.001; Fig. 5G).

Relative expression of *DWARF4* was not statistically affected by UV treatment in either Col‐0 or *uvr8‐6* petioles when compared with their respective PAR control (Fig. 5H). However, when comparing genotypes at 310 nm, relative expression of *DWARF4* was significantly higher in *uvr8‐6* (*P* = 0.01; Fig. 5H).

The UV treatment had a direct effect on expression levels of two key transcription factors, *BIM1* and *BES1*. For Col‐0, *BIM1* expression was downregulated at 310 nm but upregulated for each UV‐A treatment (*P* = 0.32; Fig. 5C). Overall, expression levels of *BIM1* were higher in Col‐0 compared to *uvr8‐6* for each UV treatment, except 310 nm, at which wavelength *BIM1* was significantly less expressed in petioles of Col‐0 (*P* = 0.036; Fig. 5F). Relative expression of *BES1* was not significantly affected (Fig. 5F).

Lastly, *BOP1* expression was affected by UV treatment in both Col‐0 and *uvr8‐6* (*P* = 0.24 and *P* < 0.001, respectively; Table S5). For uvr8‐6, expression of *BOP1* was slightly downregulated under 310 nm and 365 nm, and upregulated under 325 and 340 nm (Table S5, Fig. 5B). Relative expression of *BOP1* was higher in Col‐0 compared with *uvr8‐6* yet this was not significant for any UV treatment (Fig. 5B).

## DISCUSSION

### The classic UV‐B phenotype: Orchestrated by co‐action of UVR8, phototropin and cryptochrome

Plants exposed to different UV‐B wavelengths had neither macroscopic damage (i.e. necrotic spots or leaf curling) nor impaired photosynthetic efficiency, as measured using chlorophyll *a* fluorometry (Cunningham *et al*. 2024). It is established that non‐SIMR UV‐B leaf morphology is largely dependent on the UV‐B‐specific photoreceptor UVR8, with co‐action of cryptochromes (Hayes *et al*. 2014; Robson *et al*. 2015; Morales *et al*. 2025). The present study found that UVR8‐dependent morphological responses of *A. thaliana* leaves varied across different parts of the leaf and were further modulated through both cryptochrome and phototropin signalling. While the data presented provide new insights into fundamental responses of plants and photoreceptors under narrow UV wavelengths, they cannot be extrapolated to natural solar radiation. For example, the use of a fixed UV irradiance across treatments resulted in different biologically effective doses, with the 310 nm treatment delivering ~6.7, 6.9 and 9.6 times more biologically effective UV, respectively, compared to the 325 nm, 340 nm and 365 nm UV‐A treatments. Under natural conditions/outdoor experiments, one could expect biological doses of UV‐A to be larger than those presented here, as plants receive 10–100 times more UV‐A than UV‐B photons (dependent on latitude, season, and atmospheric conditions). However, the biological doses utilised in this study are comparable with daily doses across different latitudes in Europe, under clear skies in spring/summer, which range from 0.528–6.23 kJ m^2^ day^−1^ (Moan 2001; Aphalo *et al*. 2012; Verdaguer *et al*. 2017).

#### UV‐B specific responses of the petiole

The dwarfing of rosettes driven by UV‐B (310 nm) was largely related to a decrease in petiole length, which was the most impeded part of the leaf in all genotypes, apart from *uvr8‐6*. Petioles of UV‐B‐exposed Col‐0 were half the length of plants exposed only to PAR, a finding that extends observations from more compact plants (Robson *et al*. 2015). In agreement with Cunningham *et al*. (2024), the current study found that the decrease in petiole length was largely mediated by UVR8, as the *uvr8‐6* mutants failed to show any significant inhibition. Furthermore, in‐depth analysis found that petiole inhibition was more pronounced in a phototropin‐deficient background (*phot1phot2* as well as *phot2*) compared to Col‐0. This implies that phototropins can perceive and mediate responses to UV‐B, impeding the dwarfing response. Earlier studies showed phototropin‐mediated phototropic bending under UV‐B radiation (Vandenbussche *et al*. 2014; Vandenbussche & Van Der Straeten 2014; Jenkins 2017), confirming activity of these photoreceptors in response to 310 nm light. Thus, it is concluded that at least two photoreceptors are active in the UV‐B part of the spectrum, and that these control petiole length through antagonistic effects.

A cryptochrome‐deficient genotype (*cry1cry2*) also displayed more petiole inhibition compared to Col‐0 (Table 2). It has been shown that UV‐B perception via both UVR8 and CRYs affects photomorphogenesis (Rai *et al*. 2020; Tissot & Ulm 2020). Indeed, Rai *et al*. (2019) found that a triple mutant lacking UVR8 and both CRYs did not survive in either natural or simulated sunlight containing UV‐B. However, when just one photoreceptor was missing plants did survive. Thus, both UVR8 and CRY are active in the UV‐B part of the spectrum, exerting antagonistic effects on petiole length. While the underlying mechanism of this interaction needs to be established, it can be speculated that the absence of one/both CRYs allowed greater activation of UVR8 due to reduced competition for COP1 binding (Rai *et al*. 2020; Tissot & Ulm 2020).

#### UV‐B specific responses of the leaf blade

It is widely accepted that UV‐B exposure causes a decrease in leaf area, through reductions in leaf length and width (Robson *et al*. 2015; Jansen *et al*. 2022). The current study found that dwarfing of leaf blades is bi‐directional, involving decreases in blade length and blade width, and is influenced by different photoreceptors. In the case of Col‐0, UV‐B caused significant decreases in length and width of the leaf blades, with both parameters showing a quantitatively similar response. UVR8 modulates these responses, with UVR8‐overexpressing lines displaying reduced extension growth, reduced cell expansion, resulting in a more compact phenotype (Favory *et al*. 2009; Fasano *et al*. 2014). In line with these findings, the current study found that *uvr8‐6* plants showed the weakest inhibition response, and neither length, width, nor leaf area of *uvr8‐6* blades were significantly affected by UV‐B. Therefore, UVR8 plays a key role in the dwarfing response of leaf blades in response to UV‐B.

For petiole elongation, the UV‐B‐driven dwarfing response of the leaf blade was also highly influenced by phototropins (Table 1; Tables S3 and S4). The largest UV‐B‐driven inhibition of leaf blade length width and area was in *phot1phot2* (Table S3). Both PHOT1 and PHOT2 are known to play a role in leaf positioning, flattening and expansion; however, their potential role in UV‐B‐driven leaf morphogenesis is largely unexplored (Christie 2007; Christie *et al*. 2015). Here, in a phototropin‐deficient background, leaf blades were narrower and shorter compared to Col‐0, with PHOT2 being the main UV‐B sensor influencing leaf width. It is concluded that PHOTs play a role in controlling leaf blade development at 310 nm, and that interactions between phototropins and UVR8 appear antagonistic, although the mechanism underlying this interplay between the two pathways remains to be established.

Unlike the antagonistic interactions between UVR8 and phototropins, previous studies have shown that UVR8 signalling is enhanced in *cry1cry2* mutants (Tissot & Ulm 2020). The current study found that, compared to Col‐0, UV‐B had a smaller negative effect on leaf area of *cry1*, and blade width of *cry1* and *cry1cry2* leaves. It was concluded that cryptochromes display some activity at 310 nm and enhance UVR8‐mediated leaf blade dwarfing.

### Exploring the UV‐A phenotype and its dependence on photoreceptors

Studies of UV‐A effects on plants have yielded seemingly contradictory results (Verdaguer *et al*. 2017; Neugart & Schreiner 2018). UV‐A covers a broad wavelength range, and spectral differences as a result of using different UV‐A sources, may have distinct effects on plants.

#### UV‐A—Effects on petioles

Overall, exposure of rosettes to short UV‐A (325 nm) gave rise to morphological changes distinct from those induced by both 340 and 365 nm wavelengths. Short UV‐A caused significant dwarfing of petioles in all genotypes except *uvr8‐6*. Indeed, *uvr8‐6* had the weakest relative inhibition of petiole elongation of all genotypes, and the petioles were significantly longer than in Col‐0 (Table 2). Therefore, it is concluded that UVR8 is central to petiole inhibition in response to short UV‐A, a finding consistent with the action spectra of UVR8 (Rai *et al*. 2020; Vanhaelewyn *et al*. 2020).

The *cry1cry2* and *cry1* genotypes displayed the strongest inhibition of petiole growth at 325 nm (Table 2). This strong morphological response is consistent with *cry1cry2* showing a pronounced gene expression response to short UV‐A wavelengths compared to the wild type (Rai *et al*. 2019, 2020). Cryptochromes have been associated with control of hypocotyl length, via negative feedback loops and direct interactions with key regulators (e.g. HBI1) of key hormonal pathways, including BR and GA signalling (Küpers *et al*. 2020; Wang *et al*. 2018a). This might be the cause of the strong petiole elongation responses across UV‐A treatments in cryptochrome‐deficient genotypes *cry1* and *cry1cry2*. Interestingly, cryptochromes impede the short UV‐A dwarfing response, while UVR8 accelerates this response. This antagonistic response is consistent with previous findings which indicate that CRYs negatively regulate UVR8‐mediated gene expression in response to shorter UV‐A wavelengths (315–350 nm), and that their inactivation will allow enhanced UVR8 action (Rai *et al*. 2019, 2020).

Both cryptochromes and phototropins are necessary for petiole growth responses to longer UV‐A wavelengths. Petioles of Col‐0 were unaffected by UV‐A at 340 nm, while UV‐A at 365 nm slightly promoted growth (Table 2). However, at 340 nm, significant petiole growth promotion was observed for both *phot1* and *phot2* genotypes (not *phot1phot2*) when compared to Col‐0. Thus, PHOT1 and PHOT2 have overlapping functions of impeding petiole elongation at 340 nm. Furthermore, UV‐A at 365 nm had a significant negative effect on petiole elongation for *phot1phot2*, while *cry1* petioles were significantly more elongated compared to Col‐0 (Table 2). These data show the importance of phototropins and cryptochromes in mediating petiole elongation under long UV‐A.

#### UV‐A—Effects on leaf blades

In direct contrast to the UVR8‐mediated dwarfing observed in petioles exposed to 325 nm, there were no observed differences in leaf blade length, width or area in either Col‐0 or *uvr8‐6* lines. However, in a phototropin‐deficient background (*phot1phot2*), short wavelength UV‐A (325 nm) had a negative effect on blade length, width and area (Tables S3 and S4; Table 1). The negative effects of deficient phototropin signalling on blade elongation were also observed under 340 and 365 nm UV. Thus, it is concluded that phototropins play a role in enhancing leaf blade expansion under short and long wavelength UV‐A, consistent with their role in biomass building, leaf expansion and positioning (Christie *et al*. 2015; Kong & Zheng 2020).

The 365 nm UV had a positive effect on leaf blade area expansion in the *cry1* mutant (Table 1). This large increase is a reflection of the role of CRY1 in regulating photomorphogenic responses, including leaf elongation and expansion. In contrast, CRY2 is involved in regulating photoperiod‐dependent flowering (Giliberto *et al*. 2005; Yu *et al*. 2010; Sharma *et al*. 2014; Ponnu *et al*. 2019; Ponnu & Hoecker 2022; Khudyakova *et al*. 2024), and there was no effect of 365 nm UV on blade area in a CRY2‐deficient background. Overall, the data reveal antagonistic effects of phototropins and cryptochromes on UV‐A‐mediated leaf blade elongation.

### The petiole—A supporting structure, yet a central player

This study identified the petiole as a central player in the UV‐mediated control of the *Arabidopsis* rosette size. To gain insight in the underlying mechanism, expression of genes involved in light responses was investigated across UV‐B and UV‐A wavelengths. Remarkably, UVR8 was found to affect gene expression across the full UV spectrum, from 310 nm through to 365 nm (Fig. 5). Early studies reported UVR8 activity extending to around 320 nm (Brown *et al*. 2009; Takeda 2021). Recently, Rai *et al*. (2020) reported UVR8 activity at wavelengths up to 350 nm. The present study shows UVR8 activity at even longer UV‐A wavelengths, that is, 365 nm. It is concluded that UVR8 affects gene expression throughout the UV‐A and UV‐B spectral zones.

#### Expression of key regulatory genes under narrowband UV‐B

UVR8 enhances PIF5 protein degradation, impeding PIF5‐mediated hypocotyl elongation (Sharma *et al*. 2019; Tavridou *et al*. 2020). Here, strong expression of the PIF5 gene was noted in UV‐B exposed *uvr8‐6*, compared to Col‐0 (Fig. 5), suggesting additional control of PIF5 at the transcription level. In contrast, PIF4 gene expression was not significantly changed at 310 nm, and neither was expression of BOP1, a negative regulator of PIF4 (Zhang *et al*. 2017). Expression of both TCP1, a positive regulator of DWARF4, and of BIM1, a negative regulator of BR growth related genes (Guo *et al*. 2010; Liang *et al*. 2018; W. Wang *et al*. 2018b; Yin *et al*. 2005) was suppressed by UV‐B in a UVR8‐dependent manner. Consistently, expression of the DWARF4 gene was enhanced in *uvr8‐6*. Therefore, it is speculated that UV‐B‐driven dwarfing of petioles is associated with interactions between UVR8, PIFs and the BR signalling pathway (Wang *et al*. 2018b; Peres *et al*. 2019).

#### Expression of key regulatory genes under narrowband UV‐A

There were both elongation and gene expression responses to UV‐A involving activity of UVR8, CRY and PHOT. It is hypothesised that the UVR8, CRY and PHOT signalling pathways compete for the COP1 protein (Podolec & Ulm 2018), and this may have caused a peak in COP1 expression at 340 nm (Fig. 5A). UVR8 is predominantly active at shorter wavelengths (i.e. 310 and 325 nm), while at 365 nm, cryptochromes and phototropins play a larger role. Thus, 340 nm is a critical UV wavelength. This was also observed as wavelength‐dependent petiole elongation, which occurs at 310 and 325 nm but changes to enhanced elongation at 365 nm. Expression of PIF5 in *uvr8‐6* also peaks at 340 nm. Together with UVR8‐dependent downregulation of PIF5 in Col‐0, this suggests that PIF5 and COP1 are central to the petiole response to UV. BOP1 expression was also upregulated under all UV‐A wavelengths in Col‐0, while it was downregulated in *uvr8‐6*. BOP proteins are involved in regulation of PIF abundance and stability (Zhang *et al*. 2017), thus further emphasising the role of PIF4.

The data provide insights into possible mechanisms controlling UVR8‐, CRY‐ and PHOT‐dependent petiole responses to UV radiation. However, it is acknowledged that expression analysis at a single time point limits interpretation of this work. A full kinetic study is required, focussing on a larger subset of genes, as well as changes in accumulation of gene products and hormones.

## CONCLUDING REMARKS

This study highlights that morphological responses of *A. thaliana* leaves are wavelength‐specific, mediated by different photoreceptors, and vary across different parts of the leaf. Narrowband UV‐B induces a more compact phenotype, orchestrated by the co‐action of UVR8, phototropins and cryptochromes, with phototropins playing a particularly important role. Exposure to shorter UV‐A caused UVR8‐mediated inhibition of elongation of petioles but not leaf blades, while at longer wavelengths petiole and leaf blade elongation and expansion were mediated more by cryptochromes and phototropins. Overall, the petiole was found to be a central player in the growth response of rosettes, with multiple interactive effects between UVR8, cryptochromes and phototropins identified across all UV‐B and UV‐A wavelengths.

## AUTHOR CONTRIBUTIONS

Conceptualization: NC, GC, APM and MAKJ; Methodology: NC, GC and MAKJ; Formal analysis: NC and MAKJ; Investigation: NC and GC; Resources: MAKJ and APM; Data curation: NC, GC and MAKJ; Writing—original draft: NC and MAKJ; Writing—review and editing: NC, GC, APM and MAKJ; Supervision: MAKJ; Project administration: MAKJ; Funding acquisition: NC, MAKJ and APM.

## FUNDING INFORMATION

The financial support of Science Foundation Ireland (grant 16/IA/4418) and Irish Research Council (grant GOIPG/2023/4071) is gratefully acknowledged.

## Supporting information


Data S1.
