# Supplementary material for: Morphological responses of Arabidopsis thaliana wild type and photoreceptor genotypes to narrowband UV radiation generated by LEDs
Source: Plant Biol (Stuttg). 2025 Sep 17;27(7):1341–52. doi: 10.1111/plb.70105 (PMC12631520; doi:10.1111/plb.70105)
Supplement: Supplementary file 1 — Data S1. [file PLB-27-1341-s001.docx]

**Supplemental:**

| **Genotype** | **Affected photoreceptor** | **Sources/Donors** |
| --- | --- | --- |
| *Uvr8-6* | UVR8 | Professor Roman Ulm  (Favory et al., 2009) |
| *Phot1* | Phototropin1 | Professor Paolo Schumacher  (Schumacher et al., 2018) |
| *Phot2* | Phototropin2 | Professor Paolo Schumacher  (Schumacher et al., 2018) |
| *Phot1Phot2* | Phototropin1 & Phototropin2 | Professor Paolo Schumacher  (Schumacher et al., 2018) |
| *Cry1* | Cryptochrome1 | NASC (SALK_042397C) |
| *Cry2* | Cryptochrome2 | NASC (SAIL_763_D08) |
| *Cry1Cry2* | Cryptochrome1 & Cryptochrome2 | Professor Stephan Wenkel  (Dolde et al., 2018) |

Supplemental Table 1: Genotypes used in this study including sources and donors

| **Gene symbol** | **Gene Name** | **Forward (F)** | **Reverse (R)** | **Ref.** |
| --- | --- | --- | --- | --- |
| UBC | UBIQUITIN CONJUGATING ENZYME 9 | CTTACAATTTCCCGCTCTGC | GTTGGGATGAACCAGAAGGA | (Li et al., 2015) |
| COP1 | CONSTITUTIVE PHOTOMORPHOGENIC1 | GCCAACCACTTCATGTCTTC | ACCCACAAAGTTCTTCTCGT | TAIR / Prime3 |
| BOP1 | BLADE ON PETIOLE 1 | ACGAGCGGAAGTAGTTTGGA | ATGGTGGTGGTGGTGATGAT | TAIR / Prime3 |
| PIF4 | PHYTOCHROME-INTERACTING FACTOR 4 | GCGAGATGGACAAGTGGTTC | GTTGGTCTCTGGAGGGGATC | TAIR / Prime3 |
| PIF5 | PHYTOCHROME-INTERACTING FACTOR 5 | CAACTCCAAGTGATGTGGATG | CAATTGCATCTGACTTTGCAT | (Pham et al., 2018) |
| BES1 | Bri1-EMS Suppressor 1 | CGGAGTAGATGTGTCGGTGA | GGAACCAATCTCGTACCGGA | TAIR/Primer3 |
| BIM1 | BES1 Interacting MYC-Like protein | TGGTGACTTCTGCCGATTCT | AGCTCAGATCTGCGACTTGT | TAIR/Primer3 |
| TCP1 | TEOSINTE BRANCHED1, CYCLOIDEA, and PCF (TCP) transcription factor 1 | TGAAGGAGAAGATATGACACACC | GCTAGAACTCTGATCTACCAACA | (Zheng et al., 2021) |
| DWF4 | DWARF4 | GGTGGAAAGTGTTACCGGTG | TCCTCCAAACGGCATGTAGT | TAIR/Primer3 |

Supplemental Table 2: Primers used in this study

Supplemental Table 3: Relative change in blade length of each genotype compared to relative change in blade length of Col-0 (%)

| **Relative blade length (%)** | | | | | | | | |
| --- | --- | --- | --- | --- | --- | --- | --- | --- |
|  | **Col-0** | ***Uvr8-6*** | ***Cry1*** | ***Cry2*** | ***Cry1Cry2*** | ***Phot1*** | ***Phot2*** | ***PhotPhot2*** |
| 310 nm | -22% | -12% | -18% | -24% | -19% | -24% | -33% | **-38%^**^** |
| 325 nm | 2% | 2% | -3% | -1% | 1% | -3% | -10% | **-9%^**^** |
| 340 nm | 4% | -1% | 2% | 2% | 7% | 7% | 3% | **-3%^*^** |
| 365 nm | 5% | 6% | **13%^*^** | 1% | 0% | 12% | -1% | -6% |

Values in bold indicate responses of genotype are significantly different to those of Col-0. Level of significance denoted with asterisks where p < 0.05 (*), p <0.01 (**) or p < 0.001 (***). The heatmap shows relative changes in relative leaf blade length across genotypes, with darker green indicating stronger positive responses and darker red indicating stronger negative responses

Supplemental Table 4: Relative change in blade width of each genotype compared to relative change in blade width of Col-0 (%)

| **Relative blade width (%)** | | | | | | | | |
| --- | --- | --- | --- | --- | --- | --- | --- | --- |
|  | **Col-0** | ***Uvr8-6*** | ***Cry1*** | ***Cry2*** | ***Cry1Cry2*** | ***Phot1*** | ***Phot2*** | ***PhotPhot2*** |
| 310 nm | -21% | -14% | **-16%^*^** | -23% | **-14%^*^** | -23% | **-32%^*^** | **-35%^**^** |
| 325 nm | 0% | 0% | **-9%^*^** | -2% | 1% | -3% | -5% | **-8%^*^** |
| 340 nm | 4% | -2% | 3% | -1% | 4% | 7% | 1% | -1% |
| 365 nm | 7% | 2% | 12% | **1%^*^** | 2% | 6% | -2% | -8% |

Values in bold indicate responses of genotype are significantly different to those of Col-0 wildtype. Level of significance denoted with asterisks where *p < 0.05 (*), p < 0.01 (**) or p < 0.001 (***).*The heatmap shows relative changes in relative leaf blade width across genotypes, with darker green indicating stronger positive responses and darker red indicating stronger negative responses.

Supplemental Table 5: Relative gene expression across UV treatments and compared with the PAR control was separately assessed for both Col-0 and *uvr8-6*. Table shows those genes of interest which were significantly affected by specific treatments for each genotype.

| **Genotype** | **Gene of interest** | **Significant differences across treatments** | **P- value** |
| --- | --- | --- | --- |
| Col-0 | *TCP1* | PAR v 310  PAR v 340 nm  PAR v 365 nm | 0.001  0.003  0.007 |
|  | *PIF4* | PAR v 365 nm | 0.02 |
|  |  |  |  |
| *Uvr8-6* | *TCP1* | PAR v 340 nm  PAR v 365 nm  340 nm v 365 nm | 0.016  0.022  < 0.001 |
|  |  |  |  |
|  | *PIF5* | PAR v 325 nm  PAR v 340 nm  PAR v 365 nm | 0.007  0.007  0.018 |
|  |  |  |  |
|  | *BOP1* | PAR v 340 nm  PAR v 365 nm  310 nm v 340 nm  340 nm v 365 nm | 0.004  0.022  0.015  < 0.001 |
